# Hands-Free Authentication for Virtual Assistants with Trusted IoT Device and Machine Learning

**DOI:** 10.3390/s22041325

**Published:** 2022-02-09

**Authors:** Victor Takashi Hayashi, Wilson Vicente Ruggiero

**Affiliations:** Polytechnic School, University of São Paulo, Sao Paulo 05508-010, Brazil; wilson@larc.usp.br

**Keywords:** IoT, machine learning, smart home, privacy, continuous authentication, virtual assistant, trusted device, testbed

## Abstract

Virtual assistants, deployed on smartphone and smart speaker devices, enable hands-free financial transactions by voice commands. Even though these voice transactions are frictionless for end users, they are susceptible to typical attacks to authentication protocols (e.g., replay). Using traditional knowledge-based or possession-based authentication with additional invasive interactions raises users concerns regarding security and usefulness. State-of-the-art schemes for trusted devices with physical unclonable functions (PUF) have complex enrollment processes. We propose a scheme based on a challenge response protocol with a trusted Internet of Things (IoT) autonomous device for hands-free scenarios (i.e., with no additional user interaction), integrated with smart home behavior for continuous authentication. The protocol was validated with automatic formal security analysis. A proof of concept with websockets presented an average response time of 383 ms for mutual authentication using a 6-message protocol with a simple enrollment process. We performed hands-free activity recognition of a specific user, based on smart home testbed data from a 2-month period, obtaining an accuracy of 97% and a recall of 81%. Given the data minimization privacy principle, we could reduce the total number of smart home events time series from 7 to 5. When compared with existing invasive solutions, our non-invasive mechanism contributes to the efforts to enhance the usability of financial institutions’ virtual assistants, while maintaining security and privacy.

## 1. Introduction

Security is one of relevant emerging challenges for the Internet of Things [1,2,3]. Security attacks in daily life [4] raises user concerns about the technology maturity. The demand for resiliency against cyber attacks faced by IoT devices reveals resource limitations (e.g., energy consumption, memory, and processing), which inhibit the use of existing asymmetric cryptography solutions [5].

Major banks worldwide offer online banking to their customers to reduce costs and improve convenience of use. Online banking makes it possible for customers to check their balances and perform many financial transactions anywhere, anytime. However, emerging cybersecurity attacks make the reliance upon single-factor authentication (e.g., username/password) a growing concern for banks. By strengthening their authentication mechanisms, banks can effectively protect the confidentiality and integrity of sensitive customer data, thus avoiding financial loss and reputation damage resulting from events such as fraud and customer data disclosure [6].

**Figure 1:**
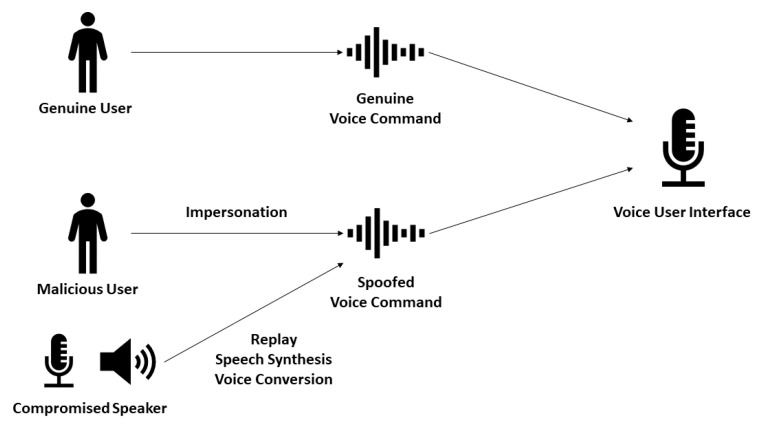
Attack examples for voice-triggered financial transactions.

A plethora of attacks impose a threat to IoT systems. Considering voice-triggered financial transactions, attacks such as impersonation, replay, speech synthesis, and voice conversion are relevant, as illustrated in Figure 1. In an impersonation attack, the adversary is a human being that tries to impersonate a genuine user’s voice; in a replay attack, prerecorded audio is played in a compromised speaker; speech synthesis is used by knowledgeable adversaries to generate artificial speech attacks; finally, voice conversion attacks take a step further, by trying to model a specific user’s voice using statistical techniques [7]. Even inaudible voice command attack feasibility has been proved in the literature [8], and existing voice recognition systems, available to Alexa and Google Home smart speaker devices, are not resilient against replay attacks [9].

The basic existing authentication mechanisms are based on knowledge, ownership, and biometrics [10,11]. When the authentication mechanism combines two or more basic authentication factors, then a multi-factor authentication is provided. Authentication methods based on “what you are” can leverage behavioral or physiological unique user characteristics [12] by using a specific digital fingerprint sensor, a frontal camera to recognize user’s face or eyes [12], or even voice recognition [9,13,14,15,16,17]. Other authentication schemes are based on the possession of something to verify user identity, such as one-time passwords by smartphone SMS, smartphone tokens, offline hardware token modules, and smart cards [18]. The methods in the “something the user knows” category rely on secret information that only the specific person should know, such as words, passphrases, and PIN (personal identification number) [11]. One relevant example in the considered scenario is the Alexa 4-pin code [19], which is a 4-digit numerical secret that the Alexa owner can set up for voice purchases.

Hands-free interactions by voice enable various use cases, such as money transfer, utility bill payment, and monitoring. Users choose to interact with smart speakers using voice commands because they perceive it as requiring less effort when compared with the smartphone alternative [20], so an invasive authentication that requires additional interaction with another device may be impractical for wide adoption.

To address these security concerns and the invasiveness in existing authentication mechanisms, this article presents a hands-free authentication scheme with a simple enrollment process. The major research contribution of this paper is that of a non-invasive authentication mechanism for financial transactions by voice, in trusted connected locations, with an additional hardware autonomous device, presenting a comparable response time and security level to existing invasive solutions; moreover, it is integrated with a method for continuous authentication, based on behavior learning in a trusted connected location (i.e., the smart home).

The article is organized as follows: Section 2 presents the state of the art; research background with usability, privacy, and security considerations are presented in Section 3. Our proposed non-invasive authentication scheme is presented in Section 4. The challenge–response protocol and its formal security analysis, using the Scyther tool, is presented in Section 5; the proof of concept is described in Section 6; the continuous authentication results are presented in Section 7. Section 8 presents a comparison of the obtained results with related work; finally, Section 9 concludes the article with final thoughts and proposed directions for future work.

## 2. Related Work

In this section, we present related work found in the literature, organized in seven categories, including single-factor and multi-factor proposed solutions.

Single-factor solutions based on voice biometrics: VoicePop is an anti-spoofing system for mobile devices. It leverages the pop noise produced by the user’s breathing while speaking close to the smartphone’s built-in microphone, which is difficult to record by an adversary beyond a certain distance. The proposed solution was validated with 18 users, 4 different mobile devices, and it could identify replay and impersonation attacks. Only accuracy-related results are available for this solution [21]. It requires no additional hardware device other than the existing mobile device used for voice interactions with the virtual assistant. A similar approach found in the literature is Two Microphone Authentication (2MA), which leverages multiple devices operating in the same area to localize and authenticate voice commands [22].

Single-factor solutions based on WiFi data: As an example of context validation, VSButton uses WiFi signals to resolve voice assistants’ vulnerabilities. These assistants are deployed in smart speaker devices, which lack physical access control, and rely solely on single-factor authentication. VSButton leverages WiFi signals to detect human motion, to authorize the smart speaker to receive voice commands [23]. WifiU solution recognizes users based on their gait. It uses two WiFi devices: one responsible for sending periodic signals (e.g., router), and one responsible for receiving signals (e.g., laptop). The channel state information (CSI) signals collected are used to identify users. The proposal was validated with a database consisting of 50 human subjects in a lab environment of 50 square meters, and it was considered to be non-invasive when compared with other approaches, such as video surveillance by cameras, and has the advantage of not requiring specific hardware, such as floor or wearable sensors [24]. A similar approach based on WiFi signals aims to capture unique human characteristics inherited from daily activities (walking and stationary). The CSI information is used to implement a deep learning model that achieved over 91% authentication accuracy with 11 subjects, considering university office and apartment environments [25].

Single-factor solutions based on trusted device: UCFL is a two-phase authentication scheme that uses IoT devices from a three-layer framework to mitigate attacks, such as replay, DoS, false data injection, and man-in-the-middle attacks. It uses physical unclonable functions (PUF) to generate unique user identifiers, and to create challenge response pairs (CRP); thus, it has a complex enrollment process. The obtained average response time was less than 150 ms, and the communication cost was 3 messages [26].

Single-factor solutions based on wearable data: EarEcho employs wireless earphones to provide user authentication with in-ear sound (unique due to the physical and geometrical characteristics of the human ear canal). It could perform continuous authentication with a set of 20 subjects with a precision of 97.57%, and a recall of 97.55% with a support vector machine (SVM) classifier [27]. Even though wearables could be used for continuous user authentication, a great challenge is to make users carry the tag inside the smart home [28].

Single-factor solutions based on sensors data: PALOT used an activity-labeled dataset from a smart home, collected with motion, temperature, item usage, and other sensors, with 24 individuals, to propose a continuous authentication scheme for IoT. It uses Markov models, ontologies, and semantic rules to authenticate users without requiring any additional device. However, the approach is heavily dependent on the deployment context [29].

Multi-factor solutions based on WiFi and voice: REVOLT is a solution that combines WiFi- and voice-based detection to mitigate replay attacks. It leverages the spectral characteristics of original and replayed voice signals, WiFi channels information, and unique breathing rate obtained from WiFi signals to test the liveness of the human voice [30]. Wivo is a voice liveness detection system designed to mitigate attacks on the voice interface of smart homes, leveraging wireless signals generated by IoT devices and received voice samples [31]. However, these systems do not support user authentication as they are focused on detecting whether the voice commands are synthetic or natural (i.e., from a person).

Multi-factor solutions based on wearables and voice recognition: Feng et al. [32] proposed VAuth, an authentication scheme for voice assistants. It is designed as a wearable security token to provide an additional channel for physical access control. It detects body surface vibrations with an accelerometer in eyeglasses, sends it by Bluetooth connection to the mobile device, and the mobile device receives the wearable data and matches it with the voice command from its microphone. This solution is resilient to replay and impersonation attacks and incurs low latency, with an average of 300 ms overhead, and it achieved 97% detection accuracy with 18 real users. However, the proposed solution enables authentication only when users are performing voice commands, not in a continuous way.

A summary of related work is presented in Table 1. None of the listed studies combine trusted device and behavior factors to perform user authentication in a non-invasive manner with a simple enrollment process.

Among the presented solutions, the ones which present accuracy and response time results are EarEcho, REVOLT, Wivo, and VAuth. There are no solutions that combine trusted device and behavior authentication factors, and none that do not require a wearable device or additional interaction other than the original voice command (i.e., non-invasive); moreover, none have a simple enrollment process. Solutions such as UCFL or PALOT may present results with more users or with a lower response time, but they are single-factor solutions. The multi-factor solutions REVOLT and Wivo have a complex enrollment process, because they are based on voice biometrics, and VAuth uses a wearable device, which we consider an invasive solution for the hands-free voice transactions scenario considered.

## 3. Research Background

### 3.1. Usability

A user’s goal when using an information system is to perform an intended task, and the authentication is the function that enables that only legitimate users perform this task using the associated system. However, the authentication procedure could be viewed as a laborious process that stands between users and their intended task, from a user’s perspective. Effective authentication design and implementation must consider usability by making it easy for legitimate users to carry out the right procedure, hard to carry out the wrong procedure, and easy to recover if a problem arises. Poor usability often results in coping mechanisms that can degrade the effectiveness of security controls [33].

User authentication must be secure but also convenient and easy to deploy and use to be widely accepted. It is possible to have several authentication schemes, as long as they are complementary and do not detract from usability. Different approaches are appropriate for distinct scenarios: speed might be prioritized for device unlocking, and memorability might be prioritized for fallback authentication, for example [34].

Considering the scope of this work, the goal of hands-free interactions is to support users in a convenient and frictionless way to carry out their daily tasks; thus, integrating a secure but invasive scheme may undermine the enhanced usability which was intended in the first place.

Feng et al. [32] proposes the use of wearable devices such as eyeglasses, earbuds, and necklaces. The authors conducted a survey with 952 participants using Amazon Mechanical Turk in the US who had previous experience with voice assistants. Using a 7-point Likert scale (from strongly disagree to strongly agree), 47% of the participants affirmed (with scores of 6 or 7) that they are willing to use a wearable device to perform authentication.

Ponticello [20] investigated the perceptions of 16 smart speaker users from Germany (15) and Italy (1) with an exploratory survey. The authors conducted of one-hour semi-structured interviews in remote and face-to-face forms. These surveys considered the following hands-free scenarios:

- Dinner party: Social gathering with friends and family, with a money transfer to a friend. The authentication was performed using Alexa 4-digit PIN;
- Utility bill payment: Utility bill payment by voice while watching TV. The authentication was performed using Alexa 4-digit PIN;
- Smart door unlocking: Unlock the front door with hands full of groceries using the voice command. The authentication was performed using Alexa 4-digit PIN;
- Check if a bill has already been paid: Check if a specific bill has already been paid by voice, while the hands are dirty after working in the garden. A variation of this scenario is to have hands dirty by preparing food in the kitchen.

These hands-free scenarios illustrate user journeys where performing an additional interaction in another device harms the usability. For example, one of the participants stated that “I think that’s impractical, because if I have to pick up a smartphone to verify myself, then I could check it right away, via an app”. The author argues that the main reason for users to interact with a voice assistant was that the interactions were effortless when compared with computers or smartphones, and that if an authentication mechanism takes away these features, the participants would be not be willing to adopt it [20].

**Figure 2:**
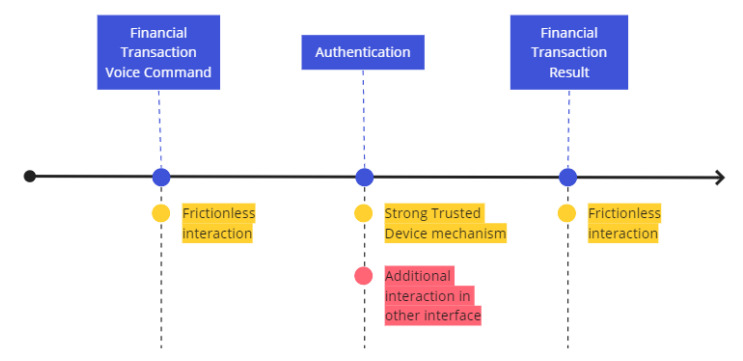
Invasive user journey with push notification in mobile device.

Consider the invasive user journey described in Figure 2, where the user initiates a hands-free financial transaction by voice using a smart speaker. However, the authentication must be preformed in another mobile device, that the user did not desire to interact with in the first place. Although the authentication may be secure by using a one-time password (OTP), the additional user interaction in another device go against the original objective of providing a hands-free interaction. The financial transaction result is provided by the smart speaker to the user in a frictionless way.

Voice biometrics was the preferred method of authentication by most of participants, but some had doubts regarding the voice recognition maturity, as illustrated by the following statement: “Currently no, not satisfied. You can tell the difference, it recognizes you by your voice, but even this recognition sometimes does not work, and I think that is very rudimentary. It’s nice that you can see that this feature is under development, but it is far from mature” [20].

The results presented by Ponticello [20] indicate that users have preference for authentication mechanisms that do not require an additional interaction with other device. However, even considering that users do not want an invasive mechanism such as a user password, they desire for these financial transactions by voice to be secure. While voice biometrics is the most preferred method, it is still not mature, according to the participants.

Therefore, we considered that our design must not require an additional interaction with another device by means of user action, and it should not rely on voice biometrics, as it still not sufficient to secure voice transactions [35].

### 3.2. Privacy

We present some privacy considerations based on existing research regarding IoT and smart speaker privacy, privacy by design, and the Brazilian and European data privacy laws.

Some open research challenges on IoT privacy are related to risk analysis, informed consent to the user (e.g., data collection and sharing), and context-aware user privacy preferences, considering the dynamic nature of IoT environments [36].

A major concern of smart speaker architectures hosted on a public cloud is privacy, as information disclosure cases have already occurred (e.g., unauthorized recording of personal conversations on Alexa). Where users demand context-aware data access control policies (e.g., who is at home and where the request comes from) [37], smart speaker user privacy perception is still at early stages (e.g., voice recognition services are available, but are not broadly used [38]).

A participant in the smart speaker user perceptions survey executed by Ponticello [20] proposed an ideal data flow to not allow Alexa or Amazon to intercept any data. The author proposes a possible technical solution to provide a direct talk between a user and their bank by decoupling the smart speaker from the Alexa cloud after the initial connection to the third party (i.e., the bank), that will be responsible for audio processing, rather than Amazon.

Privacy by design principles are applied to systems design to mitigate privacy concerns at an early stage. Gürses et al [39] discuss engineering privacy by design, which consists of principles that may be applied to mitigate privacy concerns and achieve data protection compliance by integrating these principles into the system development process. The two case studies presented followed four main steps: functional requirements analysis to assess if the functionalities are feasible and well defined, as vague or implausible descriptions may lead to solutions that collect more data than necessary; data minimization, including state-of-the-art research to evaluate which data may be minimized or whether there are alternative architectures that could contribute to the data minimization; modeling attackers, threats, and risks; multilateral security requirements analysis to consider conflicting nonfunctional requirements and constraints (e.g., integrity, availability); and implementation and testing of the design in a solution that fulfills the functionalities, while using and revealing the minimal amount of private data.

The Brazilian law LGPD (“Lei Geral de Proteção de Dados” in Portuguese or “General Data Protection Law” in free translation to English) was approved in August 2018, with its enforcement since August 2020. It describes personal data as information related to an identifiable or identified natural person; sensible personal data is described as information regarding racial or ethnic origin, political opinion, syndicate affiliation, affiliation to a religious, philosophical, or political organization, sexual life or health-related data, and genetic or biometrics data. Among others, this law has the following principles: data processing according to a finality, accorded with the data holder; free access by the data holder to data treatment information and data integrity; transparency regarding data treatment procedures and associated treatment agents; prevent damage related to personal data leakage; proof of compliance with data protection rules. However, sensible data can be treated with user’s consent for specific uses, or even without user content if it is imperative to ensure fraud prevention or security of the holder, in the identification and authentication processes in electronic systems. In addition, children’s consent is based on the consent of their legally responsible counterpart, and it foresees the need for risk and failure management for everyone who uses personal data. There is also centralized inspection by a national authority and the possibility of severe penalties in cases of non-compliance with the law [40].

Aleksanjan [41] investigated the compliance of virtual personal assistants (Amazon Alexa, Apple Siri, Microsoft Cortana, Google Assistant, Samsung Bixby) with the European Data Protection Framework, including the General Data Protection Regulation (GDPR) law, by analyzing the associated privacy policies. The results indicate that the five companies analyzed are not fully compliant with the GDPR, with Google standing out in the transparency aspect. Apple failed to inform data subjects about their rights; Amazon, Apple, and Microsoft did not adequately inform the data subjects about the purposes for processing their personal data, and relevant legal basis were not mentioned.

The GDPR was adopted in April 2016 with enforcement since May 2018, and it describes rules regarding how personal data should be processed, the subjects’ rights and the sanctions if the rules are not followed. It states that personal data must be processed lawfully, fairly, and in a transparent manner, with purpose limitation and data minimization. It also considers integrity, confidentiality, transparency, and accountability principles, conditions for consent, and specific conditions for children’s data processing [41]. The following GDPR principles may be considered:

- The purpose limitation means that data controllers should only use the collected data for specific purposes. These purposes should be explicit and determined at the time of the personal data collection;
- The data minimization principle refers to personal data being adequate, relevant, and limited to what is necessary considering the purposes for which they are processed (i.e., data controllers are only allowed to collect personal data that is necessary to fulfill the specific purpose);
- The transparency principle states that the processing of personal data should be transparent to the data subject, who must be knowledgeable about their rights and have the means to exercise it. Natural persons should be aware of the risks, rules, and rights in relation to the processing of personal data;
- The accountability principle refers to the controller being able to demonstrate compliance with the privacy principles.

We will specifically address the data minimization principle by investigating how the number of time series from a smart home impacts on the hands-free activity recognition for continuous authentication.

### 3.3. Security

**Figure 3:**
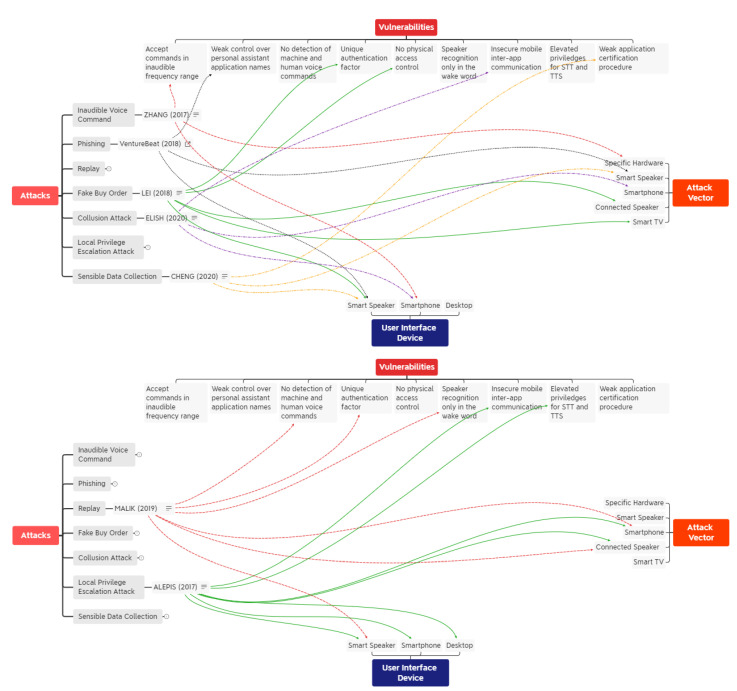
Attack relationships with Vulnerabilities, User Interface Device, and Attack Vector. Inaudible command, phishing, fake buy, collusion and sensible data collection attacks are illustrated in the top panel. Replay and local privilege escalation attacks are illustrated in the bottom panel.

In this section, we present a literature review on existing vulnerabilities and attacks in personal assistant systems. It is organized as presented in Figure 3, whose objective is to provide relationships among vulnerabilities, user interface devices, attacks, and their vectors, as described in the literature.

Existing vulnerabilities in personal assistant systems threaten the security of financial transactions using the new interface. These vulnerabilities can be related to weak authentication mechanisms, or even incomplete voice application certification procedures. User interface devices are those on which human users can initiate an interaction with a personal assistant. Some can provide additional authentication mechanisms, such as desktop and smartphone devices. The attack vector is the compromised device used by the adversary to execute an attack. For example, it is possible to use the same device which the personal assistant is integrated into, or to use another device to initiate the attack. The attacks may have financial consequences, or even characterize an invasion of privacy. Some attacks might need more knowledgeable adversaries, but some are simple to execute, such as the replay attack.

The existing authentication mechanisms, based on knowledge, possession, and biometrics, are susceptible to some threats and attacks. Something you know may be disclosed to an attacker, something you have may be lost, damaged, stolen, or cloned, and something about you may be replicated. Replay, phishing, social engineering, and man-in-the-middle (MitM) attacks could be performed by motivated attackers. For example, even a one-time password (OTP) authenticator that requires a manual entry of its output shall not be considered impersonation-resistant because the manual entry does not bind the authenticator output to the specific session being authenticated. Consider a MitM attack: an impostor verifier could replay the OTP authenticator output to the verifier and successfully impersonate the user [33].

The first attack found in the literature was the DolphinAttack [8], highlighted in red in Figure 3. Inaudible voice commands were recognized by commercial speech recognition systems, such as Siri, Google Now, and Alexa. These commands were produced by a specific hardware (amplifier and ultrasonic transducer), and the attacks were validated with experiments using smartphones from various vendors (e.g., Apple, LG, Asus, Samsung, Huawei, Lenovo). It was feasible to initiate a FaceTime call in iPhone, and to put smartphones in airplane mode using Google Now.

The second attack is fake order [23], highlighted in green in Figure 3. In this attack, the adversary could exploit smart speaker vulnerabilities to place orders in Google Express and Amazon. The vulnerabilities considered are the reliance on single-factor authentication, and no physical access control mechanism in Alexa devices. The acoustic devices used in the attack are Bluetooth speakers and smart TVs.

The third attack regards privacy concerns [42], highlighted in yellow in Figure 3. It was demonstrated that the voice application certification process is still immature: 100% of 234 Alexa skills and 39% of 381 Google actions with privacy violations were successfully certified. With no re-certification procedure, the voice application could be modified after initial certification without any additional validation, and personal sensitive information (i.e., name) could be collected in third-party servers by using children-intended Alexa skills.

Phishing attacks [43] are also feasible in voice applications, as highlighted in black in Figure 3. The certification process provide weak control over personal assistant application names, so users could activate and interact with malicious applications whose names resemble trusted voice applications.

Existing vulnerabilities in mobile devices could also be exploited if the personal assistant is deployed in a smartphone (e.g., Siri, Google Assistant). Collusion attacks can be performed using inter-app communication to perform elevated privilege actions using two or more Android applications [44]. This attack is highlighted in purple in Figure 3.

There is a dangerous combination of voice input and output permissions in Android devices and the chain of attacks from one device to another. As stated in a study found in the literature [35], even solutions such as voice recognition could not be considered a panacea, as attacks could be initiated from nearby connected devices with speakers (e.g., smartphone or Bluetooth speaker). Inter-app communication, use of microphones by using intents, and the unique coupled permission of voice input and output, are described as potential threats in Android devices. The attack that could make a malicious application take control of voice input without user acknowledgment is highlighted in green in Figure 3.

The inability to detect fake audio, and the reliance in a non-invasive unique authentication factor (e.g., voice) are some vulnerabilities that lead to replay attacks. Alexa speaker recognition system is not capable of distinguishing recorded audio from real voice, and Google speaker recognition system only performs voice verification on the wake word (i.e., “Ok Google”). If nearby devices with integrated speakers are compromised, then adversaries can record genuine voice commands and replay it afterwards, successfully performing voice replay attacks by leveraging the vulnerabilities present in the smart speaker user interface. The replay attack is highlighted in red in Figure 3.

The attacks presented in this section are by no means exhaustive. For example, there are other attacks such as LightCommands [45], which consists of a signal injection attack by converting light to sound to obtain control on Amazon Alexa, Google Assistant, Apple Siri, and Facebook Portal, at distances up to 110 meters. Another attack that may be executed without user notice is CommanderSong [46], which is an attack generated automatically by integrating voice commands and background noise into songs, difficult for human listeners to detect.

## 4. Proposed Non-Invasive Scheme

### 4.1. Design Goals

We model the existing smart home voice transactions scenario by defining the bank server, internet banking, trusted mobile, and voice user interface components.

**Definition** **1** (Bank Server—BS)**.** *The bank server is the bank authentication server which is integrated with bank back office services that effectively authorize and execute financial transactions (e.g., money transfers).*

**Definition** **2** (Internet Banking—IB)**.** *The internet banking mobile application makes banking services available to users. This application is deployed in the mobile device, and it is the existing interface for banking services.*

**Definition** **3** (Trusted Mobile—TM)**.** *A trusted mobile is a mobile application used for authentication with the bank server. The users and their trusted mobiles are associated in the enrollment phase. There is an injective relationship between an user and their trusted device (i.e., each trusted mobile is associated with an unique user, and each user is associated with an unique trusted mobile).*

**Definition** **4** (Voice User Interface—VUI)**.** *The voice user interface makes personal assistants, such as Alexa and Google Assistant, available to users. The voice commands and queries are performed by users in a frictionless manner (i.e., only the voice is needed to perform commands to voice user interfaces). The voice user interface communicates with the internet banking application in the same mobile device.*

Additionally, we consider the definitions of trusted IoT device, trusted location, and non-invasive authentication, which are fundamental building blocks of the proposed scheme.

**Definition** **5** (Trusted IoT Device—TIoTD)**.** *A trusted IoT device is a proposed specific device used with the trusted mobile to perform authentication with the bank server. It is an additional device other than the existing mobile device used for internet banking, and it is deployed on a trusted location. The users and their trusted IoT devices are associated in the enrollment phase. There is an injective relationship between an user and their trusted IoT device.*

**Definition** **6** (Trusted Location)**.** *A trusted location is a place where the genuine user visits frequently. The frequency must be at least weekly, and the trusted location for each genuine user are registered in the enrollment phase. Examples of trusted locations are workplaces and residences.*

**Definition** **7** (Non-invasive Authentication)**.** *A non-invasive authentication for a voice financial transaction command is an authentication that does not require additional interactions for the end user, nor does it require that the end user must hold a wearable device. Examples of non-invasive authentication are voice authentication and the proposed authentication performed with a trusted IoT device in an autonomous manner.*

**Figure 4:**
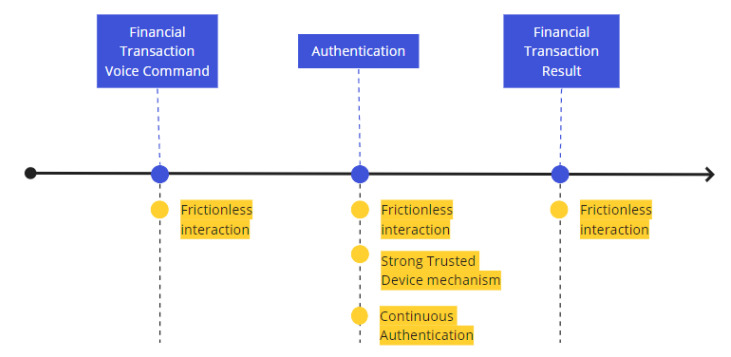
Non-invasive user journey with continuous authentication in a trusted connected location.

Considering the potential attacks and usability discussion presented, it is desirable that the proposed solution support hands-free voice transactions. Taking into account the reliance upon a trusted IoT device deployed in a trusted location, we envision the non-invasive user experience illustrated in Figure 4.

The hands-free interactions are maintained in the three steps, from the financial voice transaction voice command to its result. The non-invasive authentication is supported by a challenge–response protocol with a trusted IoT device, and a continuous authentication is performed using the behavior learned in a trusted location (i.e., in this case, the smart home).

The combination of trusted device and continuous authentication is performed in an autonomous way to support hands-free authentication, thus not requiring any additional user interactions, such as a confirmation in the mobile device.

The considered requirements are presented:

- The mechanism must provide mutual authentication;
- The novel authentication mechanism must have at least the same security level as the existing invasive authentication mechanism (i.e., smartphone token in internet banking (IB));
- The authentication mechanism must have a comparable response time with the state-of-the-art schemes found in the literature;
- The mechanism must be a non-invasive procedure. It should provide an acceptable security level, while maintaining the usability of the voice user interface (VUI).

### 4.2. Threat Model

In this article, we consider the replay and fake order attacks to the voice user interface available in a smart speaker device using a compromised nearby speaker, due to how easy it is to perform these attacks. Other attacks that require specific hardware or attacker’s physical presence were considered more complex thus performed by more knowledgeable adversaries; therefore, they are considered to be outside the scope of this study.

**Figure 5:**
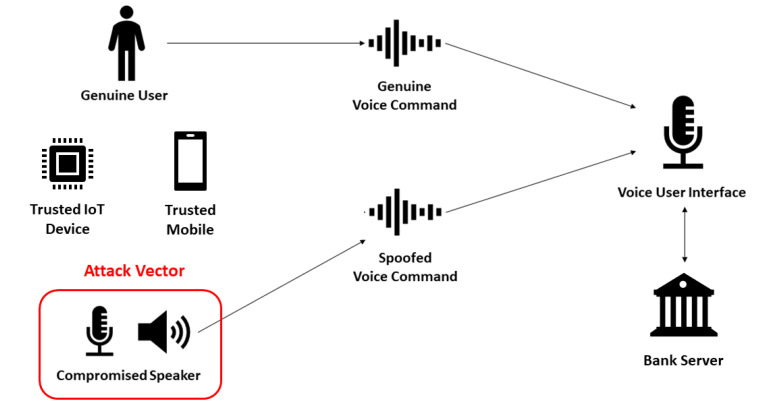
Threat model with the compromised speaker as the attack vector.

The threat model is defined below, and illustrated in Figure 5:

Adversary’s Goal: The adversary wishes to reduce the legitimate user’s balance; Adversary’s Knowledge: The adversary has access to some data samples from previous financial transactions voice commands collected from a nearby compromised speaker device (e.g., personal computer or smart TV);
Adversary’s Capability: The adversary can control the compromised nearby speaker device to play a previous voice command or an altered voice command whenever convenient. The random number used in the authentication can not be guessed by the attacker;
Adversary’s Limitation: The inter-app communication in the mobile device is considered secure (i.e., the adversary can not get the shared key in the mobile device by collusion attack [44]), as we rely in the mobile operating system security. The adversary does not have the resources to perform a massive attack to the bank server and compromise the shared keys in the bank’s possession. The trusted IoT device is considered secure, and we consider that the adversary can not steal it from the legitimate owner in the trusted location. Internal attacks to the voice user interface, such as phishing, are out of the scope of this study.

### 4.3. Assumptions and Hypotheses

We consider the following two assumptions and two hypotheses in the development of the proposed non-invasive scheme.

**Assumption** **1.** *It is desirable to not use existing mechanisms with high computational load, such as asymmetric cryptography, considering the constrained Internet of Things (IoT) devices’ performances.*

As IoT devices are constrained (e.g., energy and computing power), using asymmetric cryptography methods is not desirable in this scenario due to their high computational cost [47].

**Assumption** **2.** *Unique biometrics authentication factors, such as voice biometrics, is not enough to guarantee security for financial transactions by voice.*

It is not possible to rely solely on the voice as a single authentication factor [35]. NIST states that biometrics shall be used only as part of multi-factor authentication with a physical authenticator (something you have) [33]. Inaudible, phishing, replay, and other attacks are proved in the literature, as described in Section 3.3.

**Hypothesis** **1.** *A continuous authentication mechanism, based on behavior learning, can be based on data collected by Internet of Things devices deployed in a trusted connected location (e.g., the smart home).*

As proposed in the literature, IoT could be leveraged to provide context-aware, continuous, and non-invasive authentication services. The main benefit is related to usability, as the user do not need to carry intrusive devices or remember complex secrets. Such solution must recognize users’ behavioral patterns to validate their identity [29] and may strengthen the authentication process at the time of access request and throughout the session, without requiring additional user intervention [48].

**Hypothesis** **2.** *Performance and privacy requirements for non-invasive user authentication are achieved with edge computing architecture and privacy by design.*

Edge computing follows the guideline of bringing the computation closer to where it is needed. It can reduce the latency of requests and reduce network costs [49,50]. The privacy by design are applied in the system conceptualization to consider privacy concerns [39]. Additional principles can be found in privacy regulations, as described in Section 3.2.

### 4.4. Architecture

**Figure 6:**
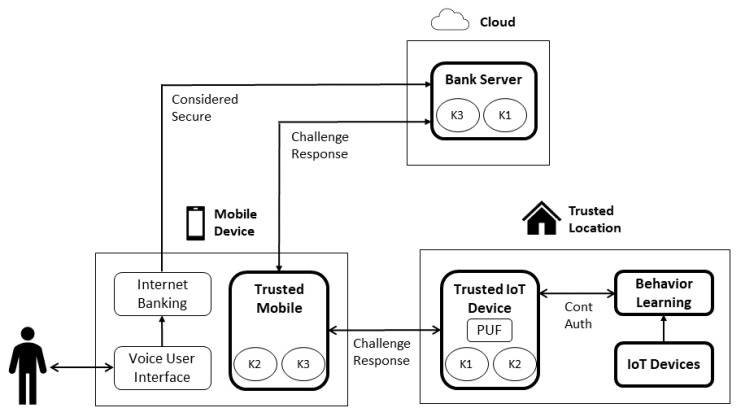
Non-invasive authentication architecture.

Figure 6 presents the proposed architecture. Consider the scenario of a financial transaction by voice. The command is captured by the voice user interface, which is integrated to various natural language processing services. When a financial transaction intent is identified, an authentication request is sent from the voice user interface to the internet banking application, deployed in the same mobile device. IB then sends the authentication request to the bank server using a secure channel, such as TLS. A challenge response protocol is performed for mutual authentication between the trusted IoT device and the bank server (deployed in the cloud) with the trusted mobile as an intermediary, based on shared keys K1, K2, and K3. The physical unclonable function (PUF) is used as input for a pseudo-random number generator used in the challenge response protocol, and not directly with challenge response pairs (CRP).

After the successful authentication, a continuous authentication is performed by leveraging the real time data collected by IoT devices for session management. If the behavior detected is different enough to a previously learned behavior, then the session is terminated. Otherwise, the session is maintained for next low value financial transactions. If the next transaction is a high value financial transaction, the challenge response protocol should be performed again.

After the user identity is validated based on the possession of the mobile device with the trusted mobile application, and the behavior biometrics from the trusted location, the bank server must provide the authentication result to the voice user interface, which can play a final voice response to the user. The scope of this work, illustrated in Figure 6, is within the highlighted blocks with thick edges (i.e., bank server, trusted mobile, trusted IoT device, behavior learning, and IoT devices).

According to different user security and privacy preferences, there is also a possibility of requiring an invasive procedure for high financial transactions, and to use or not the collected data by IoT devices. Considering the purpose limitation and transparency principles, the purposes, risks, and rights of the IoT data must be made transparent to the user prior to the possible continuous authentication deployment. The behavior learning must also support data minimization.

The inter-app communications in the mobile environment are considered secure. Attacks on inter-app communications which exploit trusted mobile operational system vulnerabilities [44] are not considered in the scope of this work. The communication between IB and BS is also considered secure. This secure communication channel could be established with transport layer security (TLS), so the messages between internet banking application and bank server are considered to be in a secure communication channel.

A final remark regards the authentication rate limit. Considering the following definition, after 5 wrong tries, the non-invasive user authentication should be disabled and a knowledge-based invasive authentication may be offered as an alternative.

**Definition** **8** (Authentication Rate Limit)**.** *NIST [33] suggests a rate limiting of 5–10 consecutive tries with a back-off time exponentially increasing.*

### 4.5. Enrollment

The enrollment process is classified as either simple or complex. Complex schemes are composed of extensive register of challenge response pairs of devices with integrated PUFs [26,51,52,53]. Our proposal is based on shared secrets, which is considered a simple enrollment process in terms of scalability and ease of management. We also do not directly use the CRP because of the associated enrollment process.

**Definition** **9** (PUF)**.** *Physical unclonable functions (PUF) are used in challenge–response protocols either directly or as sources of randomness. By leveraging their unique physical characteristics originated from the manufacturing process, strong PUFs generate large challenge response pair (CRP) spaces [54,55]. PUFs can also be used in session key generation [56].*

**Definition** **10** (Complex Enrollment)**.** *A complex enrollment process is defined as a manual and onerous operation to the end user. Some examples are the offline provisioning process of PUFs and facial and voice biometrics registration.*

**Figure 7:**
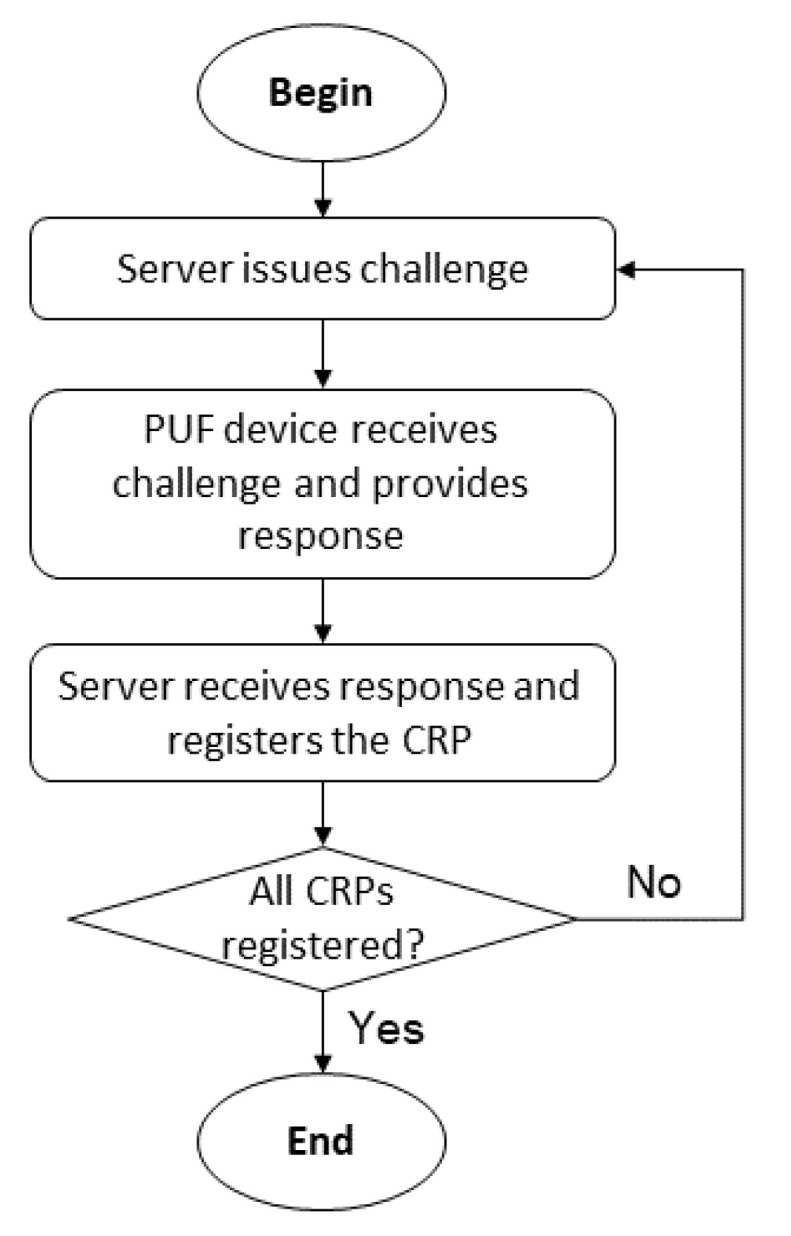
PUF challenge response pairs laborious offline registration process.

As illustrated in Figure 7, PUF-based enrollment consists of an offline provisioning procedure wherein the PUF chip is directly connected to a fog/edge device (considered a server entity). A single random serial number is the id of the PUF device and is sent together with the response to challenges issued by server. The challenge response pairs (CRP) are mapped with the serial number and sent from server to the cloud in a secure manner. For example, it might consist of a generation of 2^N^ CRP for a strong arbiter PUF of N bits (i.e., for a challenge of 16 bits, there are more than 60,000 CRPs) [57]. The direct usage of CRP generated by PUF requires that the server stores a large amount of CRP pairs, escalating proportionally to the number of devices [58].

Considering that the user already has an invasive authenticator registered with their bank (e.g., smartphone), the enrollment of the trusted IoT device can be classified as a binding of an additional authenticator at existing authentication assurance level. According to NIST [33], in this case, the user must authenticate with the existing authenticator to add the new authenticator. After successful addition, a notification should be issued to the user via independent mechanism, such as an email address previously associated with the user.

The user must register its trusted locations (according to the personal privacy preferences), trusted mobile, and trusted IoT device. The shared keys between trusted IoT device, trusted mobile, and bank server are registered in each entity. These shared keys must be at least 128 bits. The identifiers (i.e., BS—bank server; TM—trusted mobile; tIoTd—trusted IoT) are also registered.

### 4.6. Continuous Authentication

Considering the following session definition, the proposed scheme uses the continuous authentication based on smart home behavior to detect anomalous situations where the session must be terminated. The session is transparent to the user for increased usability, and the accuracy of the smart home continuous authentication influences the user experience directly.

**Definition** **11** (Session)**.** *Poor usability of frequent invasive user authentication motivates users to perform workarounds, such as cached unlocking credentials that negate the authentication freshness. A session host performs session management for increased usability and security. A session is initialized in response to an authentication event by a session subject. The session host generates a secret of 64 bits for session binding and provides it to the session subject. A session may be terminated by inactivity timeout, explicit logout event, or other events [33].*

The smart home continuous authentication module learns the usual behavior of the household based on the data collected by IoT devices deployed in this trusted location. When an user is authenticated successfully by the challenge–response protocol with the trusted IoT device, the continuous authentication begins to monitor the smart home events in real time. If the module detects an unusual behavior, it terminates the session.

As NIST [33] also states, the reauthentication procedure to prevent session termination may be performed by the presentation of a biometric authenticator, which motivated the usage of the behavior biometrics to provide continuous authentication for session management.

## 5. Challenge–Response Protocol

In this section, the authentication protocol for mutual authentication is presented. Formal security analysis of the proposed protocols is also presented.

The authentication protocol is based on the SKID3 protocol [59]. SKID3 is a 3-step protocol that supports mutual authentication, and it is suitable for devices with limitations, as stated in [60]. It is based on random numbers as the protocol nonces.

We consider three shared keys to adequate these extended protocols to support the three entities (BS, TIoTD, and TM). The key K1 is the shared key between BS and TIoTD; key K2 is the shared key between TIoTD and TM; key K3 is the shared key between BS and TM.

### 5.1. Protocol Description

The bank server generates a random number as the first challenge, associates the trusted IoT device identifier, and performs two symmetric cryptography procedures: a keyed hash with shared key K1, and another keyed hash with shared key K3 (M1). The trusted mobile receives the message, decrypts the first layer with K3, and sends the result to the trusted IoT device (M2). The trusted IoT device receives its identifier and the first challenge, generates another challenge, and provides the answer with bank server identifier with two procedures: keyed hash with K1, and keyed hash using K2 (M3). The trusted mobile receives the message, decrypts the first layer with K2, and sends the result to the bank server (M4). The bank server receives its identifier, the second challenge, and the answer to the first challenge, computes the answer to the second challenge, performs a keyed hash with K1, and sends it to the trusted mobile (M5). The trusted mobile receives the message, performs another keyed hash with K2, and sends the result to the trusted IoT device (M6). The trusted IoT device must decrypt the final message with K2 and K1 to verify bank server’s identity proof.

Consider the following notation for the protocol description:

- TM—trusted mobile;
- BS—bank server;
- rBS—random number generated by BS;
- rTM—random number generated by TM;
- bs—BS identifier;
- tm—TM identifier;
- K1—the shared key between TM and BS;
- hK1()—hash function with shared key K1;
- “,” denotes concatenation;
- TIoTD—trusted IoT device;
- rTIoTD—the random number generated by TIoTD;
- tIoTd—TIoTD identifier;
- K3—the shared key between TM and BS;
- hK3()—hash function with shared key K3.

The protocol messages are as follows:

M1: TM ← BS: hK3(hK1(rBS,tIoTd))
M2: TIoTD ← TM: hK1(rBS,tIoTd)
M3: TIoTD → TM: hK2(hK1(rTIoTD,rBS,bs))
M4: TM → BS: hK1(rTIoTD,rBS,bs)
M5: TM ← BS: hK1(rTIoTD,rBS,tIoTd)
M6: TIoTD ← TM: hK2(hK1(rTIoTD,rBS,tIoTd))

**Figure 8:**
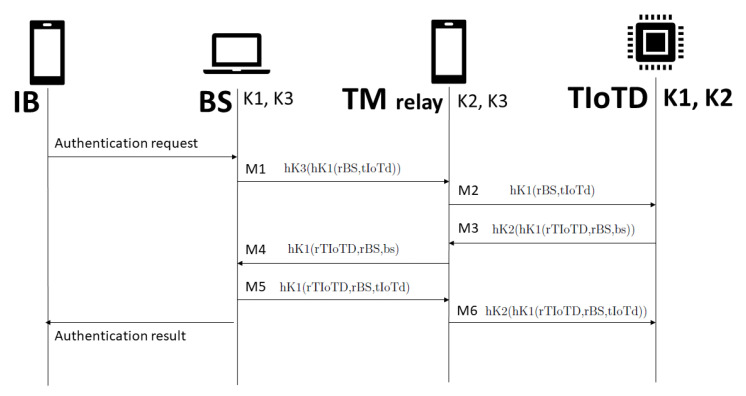
Sequence diagram of proposed authentication procedure.

The proposed authentication scheme is illustrated in Figure 8.

### 5.2. Formal Security Analysis

An automated security analysis using Scyther tool is presented for the proposed protocols for trusted mobile and trusted IoT device scenarios.

Scyther is an open-source tool that allows verification and analysis of security protocols. It is based on a formal semantics of security protocols to analyze different classes of attacks, and possible protocol behaviors [61].

Scyther provides a graphical user interface, a Python command line interface, and can be used in Windows and Linux operational systems. In the unbound mode, Scyther can output proof and attack trees, and in the bound mode, Scyther states that no attacks exist within a certain bound, or showcases some identified attacks. Its input language resembles C/Java-like syntax, and allows the modeler to describe protocols by defining a set of roles, which are defined by a sequence of events [62].

Scyther allows the verification of claims related to authentication properties of analyzed protocols. These properties are defined in Reference [63]: aliveness, weak agreement, non-injective agreement, and injective agreement.

**Definition** **12** (Aliveness)**.** *After entity A completes a run of the protocol, if another entity B is apparently active, then the protocol guarantees aliveness of entity B to entity A.*

**Definition** **13** (Weak Agreement)**.** *If the protocol guarantees aliveness of entity B to entity A, and if the protocol also guarantees aliveness of entity A to entity B, then a weak agreement is guaranteed between entities A and B.*

**Definition** **14** (Non-injective Agreement)**.** *A protocol guarantees an initiator A non-injective agreement of another agent B on a set of data D if entities A and B have weak agreement, and the two agents agreed on the data values present in D.*

**Definition** **15** (Injective Agreement)**.** *A protocol guarantees an initiator A injective agreement of another agent B on a set of data D, if entities A and B have non-injective agreement, and each protocol run of A corresponds to a unique run of B. This one–one relationship may be important in financial protocols.*

The proposed protocol for the trusted IoT device scenario was modeled based on existing Scyther models [64] of the ISO 9798 standard for entity authentication, which were used for the conception of SKID3 protocol [59].

The Scyther tool identified that challenges must also be protected, so that entities respond only to valid entry challenges. Three keys were necessary: K1 for trusted IoT device and bank server, K2 for trusted mobile and trusted IoT device, and K3 for trusted mobile and bank server.

The properties secrecy, aliveness, weak agreement, non-injective agreement, and injective agreement could be proved using the Scyther tool for the extended SKID3 protocol with the three shared keys.

All protocol models are available online under GPL-2.0 License (https://github.com/vthayashi/scyther-auth, accessed on 11 December 2021).

## 6. Trusted IoT Device

A proof of concept is implemented for the proposed protocol. It is composed of a mobile application for the authentication module, a server for bank server emulation, a web application for voice user interface emulation, and an embedded application for the trusted IoT device.

This proof of concept is designed to have the same security level as state-of-the-art, non-invasive, PUF-based authentication, with the benefits of supporting a non-invasive authentication with a simple enrollment process, and the use of PUF to improve nonces randomness.

### 6.1. Methods and Materials

**Figure 9:**
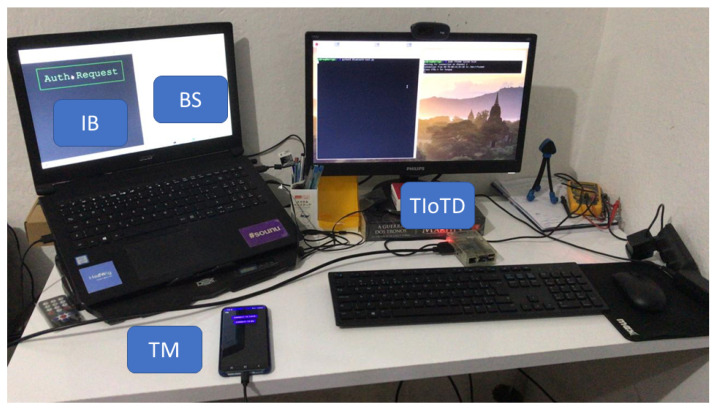
Testbed for trusted IoT device proof of concept.

The mobile application TM was developed for Android devices in Java, the BS server was developed in Python, and the webpage for the VUI was developed in HTML/Javascript using available libraries for Android [65,66] and Python [67,68]. The trusted IoT device is developed based on existing python libraries [67,68,69], and integrated Bluetooth 4.1 support for the Raspberry Pi 3 (https://www.cnet.com/, accessed on 11 December 2021). The devices used were an Android smartphone Samsung S20, a Windows laptop with 8GB RAM, a router with 802.11 communication, and a Raspberry Pi 3, as illustrated in Figure 9.

The proof of concept was executed in a local environment, with websockets communication, over WiFi and USB communication. Shared keys of 136 bits were used for the keyed hash (HMAC) with SHA256, and for the version with AES-256 symmetric key encryption. All the code and response time results for the proof of concept are available online under a GPL-3.0 License (https://github.com/vthayashi/SKID3-PoC, accessed on 11 December 2021).

### 6.2. Tests and Implementation

The proof of concept was evaluated with the four following tests:

1. Correct shared keys;
2. Correct shared key K1, correct shared key K2, and wrong shared key K3;
3. Correct shared key K1, wrong shared key K2, and correct shared key K3;
4. Wrong shared key K1, correct shared key K2, and correct shared key K3.

**Figure 10:**
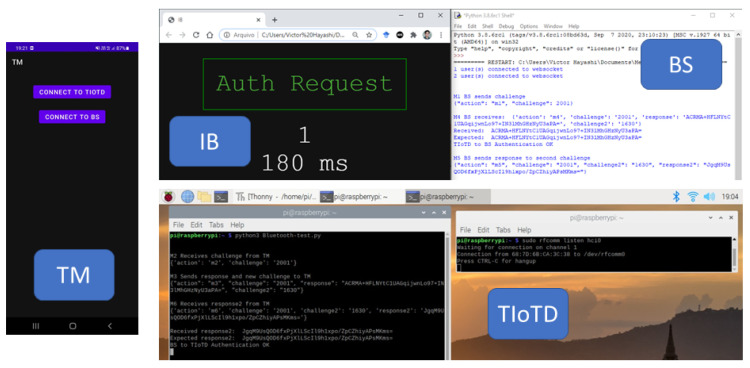
Proposed protocol with SHA-256 hash.

**Figure 11:**
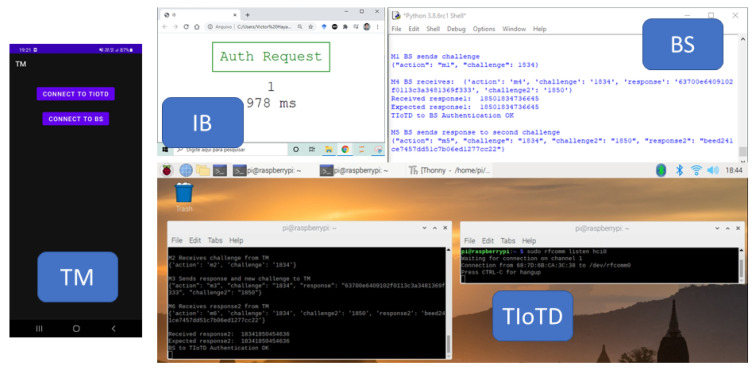
Proposed protocol with AES-256.

The tests were successfully executed, as shown in Figure 10 and Figure 11, with the proposed protocol using SHA-256 and AES-256, respectively.

### 6.3. Performance Analysis

The response time for the extended SKID3 protocol was obtained experimentally. A total of 1000 authentication requests are performed for each scenario, with a 2-s interval between requests. A normal distribution was assumed for experimental results; thus a confidence interval was obtained with a confidence level of 95%.

Considering a normal distribution for SHA-256 hash experimental results, sample size of 1000, computed standard deviation of 172.18 ms, and a significance level of 5%, we have an average response time of 392.37 ms ± 10.67 ms (i.e., from 381.70 ms to 403.04 ms), with a confidence level of 95% with serial communication (USB).

Considering a normal distribution for SHA-256 hash experimental results, sample size of 1000, computed standard deviation of 189.19 ms, and a significance level of 5%, we have an average response time of 542.76 ms ± 11.73 ms (i.e., from 531.04 ms to 554.49 ms), with a confidence level of 95% with wireless communication (WiFi).

Considering a normal distribution for AES-256 experimental results, sample size of 1000, computed standard deviation of 146.83 ms, and a significance level of 5%, we have an average response time of 383.76 ms ± 9.10 ms (i.e., from 374.66 ms to 392.86 ms), with a confidence level of 95% with serial communication (USB).

We have an average response time of 578.96 ms ± 11.99 ms (i.e., from 566.97 ms to 590.95 ms) with a confidence level of 95% with wireless communication (WiFi), with a normal distribution for AES-256 experimental results, sample size of 1000, computed standard deviation of 193.46 ms, and a significance level of 5%.

## 7. Continuous Authentication

The interested reader may consider previous work, which describes in detail the behavior learning in a smart home [70]. In this article, we focus on presenting how such a behavior factor may be integrated into continuous authentication to support session management (i.e., session beginning and end) in the proposed scheme. Our approach consists of leveraging energy consumption data collected by IoT devices to detect hands-free activity detection, as further explained.

One additional requirement is related to the data minimization principle described in Section 3.2. As the personal data must be relevant and limited to what is necessary, we verify how granular the collected data should be to enable the recognition of hands-free activity detection.

### 7.1. Considered Scenarios

We consider two scenarios for hands-free voice interactions. The first one happens whenever the user does not have IoT devices in the connected trusted location, or in the initial learning phase of the behavior learning model. In this situation, the user can activate or deactivate the hands-free authentication alternative, by using an invasive authentication method (e.g., token in the mobile device). If the user knows that they might perform a financial transaction by voice in the near future, it is possible to activate the hands-free authentication in advance, and disable it after the financial transaction has been performed, in a similar way to how users unlock their virtual credit cards in advance. This initial manual phase provides data labeling (i.e., the timestamps the hands-free financial transactions are performed), which is used to automatize the hands-free activity detection in the second scenario.

The second scenario is the non-invasive authentication for financial transactions by voice, with automated session management supported by hands-free activity detection. The user can specify in which contexts he/she wishes to activate the hands-free interactions automatically. Whenever the user is in a hands-free context and performs a financial transaction by voice, the non-invasive authentication with the trusted IoT and mobile devices is performed for increased usability. As described in Section 3.1, some hands-free scenarios with financial transactions are money transfer in dinner party with friends, and payment while watching TV. Some works found in the literature investigate daily activity recognition and forecast in smart homes. It is possible to classify some of these daily activities as hands-free activities: cooking, eating, reading, washing dishes, and watching TV from [71], and cooking, eating, relaxing, and washing dishes from [72].

**Figure 12:**
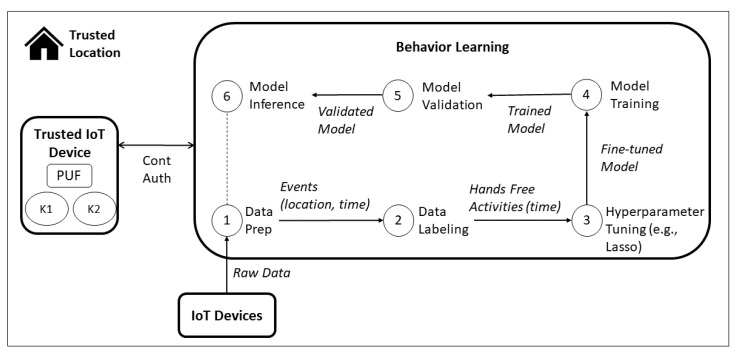
Hands-free activity detection proof of concept.

The proof of concept for hands-free activity detection in smart homes is presented in Figure 12. The raw data is collected by the IoT devices installed in the trusted location. In the data preparation step, the data from different devices is aggregated in a dataset consisting of events that occurred in specific time slots, and in a specific location inside the household (e.g., higher energy consumption in the kitchen in the first hour of a workday). Based on the events metadata and calendar of the smart home inhabitants, the events are labeled based on a subset of hands-free activities (i.e., watching TV, eating lunch and dinner). With a relevant dataset with data of at least one month, the hyperparameter tuning, model training, and validation steps are performed. Based on the existing promising results of daily activity recognition with support vector machines (SVM) [73], we selected this machine learning model to develop our proof of concept. If the model has an accuracy of over a specified threshold, then the model is deployed, and made available to detect hands-free activities in real time. This model is integrated with the proposed scheme to allow automatized session management.

### 7.2. Testbed Data Collection

The smart home testbed is the same household with four inhabitants presented in a previous work [70], but using data collected with energy monitoring sensors instead of the light and motion sensors. The data was collected from June 2021 to August 2021, a total of 2 months.

The smart meter used is the prototype presented in [74]. The smart meter and smart plugs used were based on the previous works on data collection using the ESP8266 development board [70,75]. In this work, we will use the consumption of the kitchen and living room household sector collected using the smart meter, and granular data collected from the kitchen (air conditioner, electric rice pan, and electric oven) and the living room (home office station, light bulb, TV) using some smart plugs. A total of 7 energy monitoring sensors are used, resulting in a dataset of hour granularity, 7 time series, and 1683 rows.

### 7.3. Proof of Concept

In the activity labeling step, we considered a fixed time window of one hour, the features of the most frequent event in the current window, a subset of the features used in [76]. Additionally, the day of week is included as a feature, based on Reference [77]. The resulting 9 features are: the 7 energy consumption time series, the weekday, and the hour. The hands-free activities of the resident that works daily in the living room home office station were labeled manually (i.e., 1 if hands-free activity, 0 if not). Most of the events are related to lunch break, and watching TV after the work schedule (usually at night).

The analysis using the Lasso Regression Model from the Python sklearn library [78] showed that the most important features are the kitchen electric oven, the living room TV, and the living room home office station. The SVM model tuning was performed using the GridSearchCV from sklearn, with 5-fold cross validation to optimize hyperparameters such as kernel, gamma, and degree (where applicable), using the f1-score metric. The dataset (1683 elements) was partitioned into the training dataset (70%, i.e., 1178 elements) and the test dataset (30%, i.e., 505 elements).

The experiments covered a total of 4 scenarios to investigate which time series must be used for the hands-free activity recognition. Scenario A includes the 7 time series in kitchen (air conditioner, electric rice pan, and electric oven), living room (home office station, light bulb, TV), and the household sector of the living room and kitchen appliances. Scenario B consists of 5 time series in the kitchen (electric rice pan and electric oven) and living room (home office station, light bulb, TV). Scenario C consists of the 3 most important features, according to the Lasso Regression analysis: the kitchen electric oven, the living room TV, and the living room home office station. The last case (scenario D) consists of kitchen appliances: the air conditioner, electric rice pan, and electric oven.

**Figure 13:**
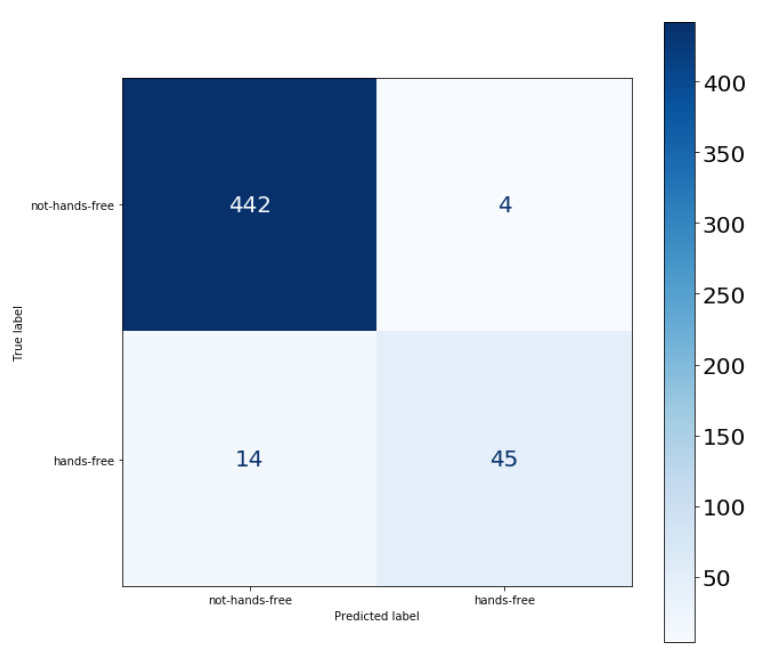
Scenario A confusion matrix for hands-free activity recognition.

The recall metric is specially important to understand how many relevant hands-free activities were classified correctly when compared to the total of hands-free activities, as illustrated in the confusion matrix of scenario A in Figure 13. The results of the 4 scenarios are presented in Table 2 considering the accuracy and recall metrics with 5-fold cross validation. The accuracy results might be compared with the general activity recognition of the SVM model of 91.52%, found in the literature [73].

## 8. Discussion

### 8.1. Assumptions and Hypotheses

Assumption 1 states that it is desirable to not use existing security mechanisms with high computational load, considering the IoT devices’ constraints. The proposed challenge–response protocol is based in the hash function SHA-256, and symmetric encryption with AES-256. Even though the SHA-256 version does not require decryption, as the AES-256 does, both versions of the 6-message protocol are based on light security mechanisms, and provide mutual authentication with a simple enrollment process.

As specified in Assumption 2, using the voice biometrics as an unique authentication factor is not enough to guarantee security for voice-triggered financial transactions. Our solution combines the trusted device paradigm (i.e., associated with “what you have”) and smart home behavior to provide a non-invasive authentication mechanism.

The continuous authentication mechanism based on data collected by IoT devices could be achieved by a hands-free activity recognition based on energy consumption data from a smart home testbed. Thus, Hypothesis 1 could be proved successfully, considering the smart home as a trusted connected location.

The performance requirements for non-invasive user authentication were achieved by relying on the local websockets communication between the trusted mobile and trusted IoT device. Additionally, the context information (i.e., the presence of the trusted mobile in the trusted connected location, both associated with the same user) was employed in our solution. Therefore, the performance aspect of Hypothesis 2 was proved successfully, based on the results of the trusted IoT device proof of concept.

As for the privacy requirements associated with Hypothesis 2, the results observed in Table 2 show that the hands-free activity recognition model performed better in the scenario B with fewer time series when compared with scenario A. Considering the data minimization principle defined in Section 3.2, a feature engineering process could be employed to reduce the number of time series to the ones which are relevant to the specific task of hands-free activity recognition. However, it is essential that such feature selection must consider time series from different smart home rooms, as one may infer by comparing the recall metric from scenarios C and D.

### 8.2. Known Limitations

Even though behavioral biometrics are suitable for the non-invasive authentication scenario, this kind of biometrics are less likely to express authentication intent because they do not need a specific action of the end user [33]. Another shortcoming of the behavioral biometrics in the smart home scenario is that the behavior learning proposed solution is highly dependable on the deployment context (i.e., which IoT devices are available in each smart home).

If an opponent compromises a trusted mobile from a specific user, no other user gets compromised. This feature decreases substantially the potential attacks scalability. Additionally, if our scheme is used with different bank server identifiers for each user, than there is less risk involved if this secret were also to be compromised.

The main vulnerability in our scheme is the shared key capture in the mobile device, as the mobile device is a general use device and could be subject to other attacks. Properly protecting the shared keys in the trusted IoT device is also of foremost importance, considering the possibility of physical attacks in this devices (e.g., side-channel attacks). We consider that obfuscation, key splitting, and secure multi-party computation mechanisms may help to enhance the security of our solution.

Another issue is the potential threat of quantum computing in the existing systems that supports financial services. Novel technologies that enable innovations, such as the Blockchain for decentralized financial transactions, face this quantum computing threat as well [79]. Even though our proposed hands-free authentication does not use public key encryption that is threatened by the quantum computers [80,81], it is relevant to include a discussion regarding this issue.

We may consider the Bitcoin scenario as an example of how the quantum computing threat can be solved. Bitcoin is a decentralized cryptocurrency that uses the Blockchain technology to perform a consensus without the need to rely on a trusted third party [82]. However, the most vulnerable aspect in the case of Bitcoin is the classical signature scheme [83], which is not used in our proposed hands-free authentication scheme. As far as the authors are concerned, Ikeda [84] is the first to solve the problem of double spending by using quantum teleportation [85]. The quantum teleportation is a method to transport quantum information to another area [82] related to the quantum communication research field. Ikeda [82,84] also uses the quantum digital signature schemes of Gottesman and Chuang [86], given by a quantum one-way function.

In the case of the hands-free authentication proposed in this work, we use an additional authentication factor based on behavior, which is independent of the cryptography used. The trusted device authentication factor rely on symmetric algorithms and hash functions that are relatively resistant to quantum computers [80,81].

According to NIST, the impacts of large scale quantum computers on AES and SHA algorithms are larger outputs for hash functions and larger key size for symmetric encryption [81]. It is possible to follow these guidelines and additionally investigate if a post quantum cryptography algorithm may be applied in our scheme. However, such investigation must consider post-quantum cryptanalysis [87] and proper evaluation [88].

Cheng et al. [89] developed an Assembler implementation of the SHA-512 hash algorithm for the ATmega 8-bit AVR microcontrollers with 128 kB flash memory and 4 kB RAM. This version of the SHA512 hash algorithm is comparable to the SHA256 implementation of Balasch et al. [90] considering short messages of 500 bytes each. Therefore, it is feasible to use the SHA512 in our scenario considering the constraints of IoT devices to make the proposed scheme resilient to quantum computers.

### 8.3. Comparison with Related Work

As shown in Table 3, the proposed solution has an accuracy of 97%, comparable to REVOLT [30] and VAuth [32]. However, REVOLT has a complex enrollment process because it is based on biometrics and behavior authentication factors, which require training time or specific biometric registration, and VAuth is an invasive solution, according to Definition 7, which defines wearable-based solutions as invasive.

The response time of 383 ms presented by our proof of concept with the SHA-256 version is comparable to VAuth and Wivo [31]. Still, Wivo relies only on the behavior authentication factor entirely thus it has a complex enrollment process because of its training phase. UCFL [26] presents the best response time, though it also relies in an unique trusted device authentication factor.

Even though our approach was evaluated with fewer users than the related work, we have validated in a real-world setting (i.e., “in the wild”), with no control over the inhabitants’ routine in the 2-month data collection period. Moreover, we have performed activity and person recognition in a multi-user scenario with 4 persons. VSButton [23] recognizes activities, but not who is performing them. Wivo performs voice liveness of certain persons, but does not recognize the associated activity, and it was validated with two users in the multiple user scenario [31]. PALOT relied on a dataset from an apartment where participants were asked to perform certain daily activities while interacting with the deployed sensors, so it presented a certain degree of control over the inhabitants activities [29]. WifiU conducted experiments to collect gait data in a typical laboratory with 50 square meters area, which is a controlled environment [24].

Considering that the multi-user scenario is a challenge for smart home algorithm development [91,92], and that it is difficult to implement a granular access control for smart speakers inside multi-user environments, we advocate that our approach helps to close this research gap, by presenting a way to perform hands-free activity recognition from a specific person. As shown in Table 2, it relies on device-level energy consumption data from two different environments for acceptable accuracy and recall results.

A comparison with third-party metrics for authentication is available in Table 4. This framework includes security, deployability, and usability aspects, so it is suitable for analyzing the proposed authentication scheme in general [93]. We chose the categories that apply to our hands-free authentication scenario: effortless to remember, nothing to carry for the end users, easy to learn, and infrequent errors under the usability aspect. Under deployability, we assess if the cost is negligible per user. We also analyze whether the mechanism is resilient to leaks from other verifiers, whether it is resilient to theft, and whether it requires explicit consent.

The 4-digit spoken PIN used in Alexa has high usability and deployability, but weak security. The mobile token is secure but invasive, thus lacking in usability, and the wearable solution requires the users to carry an additional device, with considerable cost per user. Voice and behavior biometrics are not yet mature, with frequent errors, and they face the issue that a leak from another verifier may compromise the factor entirely (i.e., the revocation is limited). We use the behavior factor as a secondary factor to identify hands-free scenarios and reduce the potential attack window, and we rely on the autonomous trusted device to not require explicit consent, which is a trade-off between security and usability. However, the cost is not negligible per user, and the case of trusted device theft is not considered within the scope of this work.

Entrophy is also an essential aspect for user authentication. Considering other authentication schemes presented in the literature, displayed in Table 5, the trusted autonomous device used in the proposed scheme provides a comparable security.

To the best of the authors’ knowledge, our proposal is one of the first works to combine trusted device and behavior factors to perform user authentication in a non-invasive manner with a simple enrollment process. Our mechanism supports mutual authentication, with a comparable security level to the existing invasive authentication mechanism, and it presents a comparable response time with state-of-the-art schemes found in the literature.

The proposed solution allows non-invasive authentication for financial transactions by voice while the user is performing hands-free activities in a trusted connected location (i.e., the smart home). The architecture considers using the PUF in the random number generator instead of directly application of challenge–response pairs, which would result in a complex enrollment process. In addition, it integrates the data minimization principle in the behavior learning process to respect the user privacy.

## 9. Final Considerations

Considering financial transactions by voice commands to personal assistants, we proposed a non-invasive mutual authentication protocol based on trusted IoT devices and hands-free activity recognition in a smart home.

Formal security analysis with the Scyther tool guided the definition of the extended versions of existing light protocols to be used in the non-invasive authentication scheme.

The first proof of concept was developed for the Android operational system, integrated with a bank server emulated in Python, with websockets communication, over a local WiFi network. It also had an authentication module in a trusted IoT device, implemented in a Raspberry Pi 3.

The second proof of concept presented how it is possible to provide hands-free activity recognition based on energy usage data collected by smart meter devices. It also employed different scenarios to investigate which subset of time series features is necessary to maintain acceptable recall and accuracy results, considering the data minimization principle.

As future work, the number generator for the trusted IoT device with physical unclonable functions (PUFs), to provide better nonces for the challenge–response protocol, could be evaluated experimentally. The dynamic random access memory (DRAM), PUF-based key generation found in the literature [97] is considered for future PUF design. Chen et al.’s solution uses intrinsic sensors available in commodity devices, and provided a proof of concept for Raspberry Pi. A relevant work for random number generator presented a proof of concept with an additional static random access memory (SRAM) and a Raspberry Pi device [98]. We also consider the RC-PUF, which is based on additional passive components (i.e., resistors and capacitors) [99].

Another research opportunity is to validate the user’s context in a more granular way, by verifying which room a certain person is in, based on direct communication between the trusted mobile and trusted IoT devices (e.g., Bluetooth). Another research opportunity is to use obfuscation, key splitting, and secure multi-party computation mechanisms to enhance the security associated with key management in trusted mobile and trusted IoT device. Additional security validations may be performed with ProVerif [100].

## Figures and Tables

**Table 1:** Related work summary.

Solution	Trusted Device	Biometrics	Behavior	Invasive	Enrollment	Users	Accuracy	Response Time (ms)
VoicePop [21]	No	Yes	No	No	Complex	18	90%	-
2MA [22]	No	Yes	No	No	Complex	-	84%	-
VSButton [23]	No	No	Yes	No	Simple	-	-	-
WifiU [24]	No	Yes	Yes	No	Complex	50	92%	-
Shi et al. [25]	No	No	Yes	No	Complex	11	92%	-
UCFL [26]	Yes	No	No	No	Simple	-	-	150
EarEcho [27]	No	Yes	No	Yes	Complex	20	95%	1000
PALOT [29]	No	No	Yes	No	Complex	24	70%	-
REVOLT [30]	No	Yes	Yes	No	Complex	10	97%	1100
Wivo [31]	No	No	Yes	No	Complex	5	96%	320
VAuth [32]	Yes	Yes	No	Yes	Simple	18	97%	300

**Table 2:** Comparison of the 4 scenarios to analyze which time series are necessary for hands-free activity recognition.

Scenario	Number of Time Series	Kitchen	Living Room	Accuracy	Hands-Free Recall
A	7	yes	yes	95.76%	76%
B	5	yes	yes	97.03%	81%
C	3	yes	yes	97.79%	80%
D	3	yes	no	92.11%	37%

**Table 3:** Comparison of proposed solution with related work.

Solution	Trusted Device	Biometrics	Behavior	Invasive	Enrollment	Users	Accuracy	Response Time (ms)
VoicePop [21]	No	Yes	No	No	Complex	18	90%	-
2MA [22]	No	Yes	No	No	Complex	-	84%	-
VSButton [23]	No	No	Yes	No	Simple	-	-	-
WifiU [24]	No	Yes	Yes	No	Complex	50	92%	-
Shi et al. [25]	No	No	Yes	No	Complex	11	92%	-
UCFL [26]	Yes	No	No	No	Simple	-	-	150
EarEcho [27]	No	Yes	No	Yes	Complex	20	95%	1000
PALOT [29]	No	No	Yes	No	Complex	24	70%	-
REVOLT [30]	No	Yes	Yes	No	Complex	10	97%	1100
Wivo [31]	No	No	Yes	No	Complex	5	96%	320
VAuth [32]	Yes	Yes	No	Yes	Simple	18	97%	300
Proposed Solution	Yes	No	Yes	No	Simple	4	97%	383

**Table 4:** Comparison of proposed solution with related work considering third-party metrics.

Solution	Usability				Deployability	Security		
	Memorywise- Effortless	Nothing- to-Carry	Easy- to-Learn	Infrequent- Errors	Negligible-Cost- per-User	Resilient-to-Leaks- from-Other-Verifiers	Resilient- to-Theft	Requiring-Explicit -Consent
4-digit spoken PIN	yes	yes	yes	yes	yes	no	yes	yes
Mobile Token	yes	no	yes	yes	yes	yes	yes	yes
Voice Biometrics	yes	yes	yes	no	yes	no	yes	no
Wearable	yes	no	yes	yes	no	yes	no	no
Behavior Biometrics	yes	yes	yes	no	yes	no	yes	no
Proposed	yes	yes	yes	yes	no	yes	no	no

**Table 5:** Entrophy comparison of proposed solution with related work.

Authentication	Entrophy (Security Bits)	Source
Voice	30	[94]
Spoken Password	46	[95]
Face Image	47	[95]
Fingerprints	69	[95]
Binary Face Template	75	[96]
Autonomous Device	128	This work
Iris	288	[95]
